# Bibliometric Examination of Global Scientific Research about Carbapenem-Resistant *Acinetobacter Baumannii* (CRAB)

**DOI:** 10.3390/antibiotics12111593

**Published:** 2023-11-04

**Authors:** Himanshu Jangid, Deepak Kumar, Gaurav Kumar, Raj Kumar, Narsimha Mamidi

**Affiliations:** 1 Department of Microbiology, School of Bioengineering and Biosciences, Lovely Professional University, Phagwara 144411, India; himanshu.12116762@lpu.in; 2 Department of Chemistry, School of Chemical Engineering and Physical Sciences, Lovely Professional University, Phagwara 144411, India; deepak.23418@lpu.co.in; 3 Department of Pharmaceutical Sciences, University of Nebraska Medical Center, Omaha, NE 68105, USA; rk7410@gmail.com; 4 Wisconsin Center for NanoBioSystems, School of Pharmacy, University of Wisconsin-Madison, Madison, WI 53705, USA

**Keywords:** carbapenem-resistant *Acinetobacter baumannii* (CRAB), MDR, XDR Gram-negative bacteria/bacilli, bibliometric analysis, thematic mapping

## Abstract

This review paper presents a comprehensive bibliometric analysis of the global scientific research pertaining to carbapenem-resistant Acinetobacter baumannii (CRAB) from the years 1996 to 2023. The review employs a systematic approach to evaluate the trends, patterns, and collaborative networks within the CRAB research landscape, shedding light on its substantial global health implications. An analysis of the Scopus database reveals that the earliest publication within the CRAB research domain dates back to 1996. By conducting a meticulous examination of publication output, citation trends, author affiliations, and keyword distributions, this paper provides valuable insights into the evolution of research themes and the emergence of new areas of interest concerning CRAB. The findings of this bibliometric analysis prominently feature the most influential author within this field, namely, Higgins PG, who has contributed a remarkable 39 documents to CRAB research. It is noteworthy that China leads in terms of the quantity of published research articles in this domain, whereas the United States occupies the foremost position about citations within the CRAB research sphere. Furthermore, a more profound exploration of the data yields a heightened understanding of the current status of CRAB research, emphasizing potential avenues for future investigations and underscoring the imperative need for collaborative initiatives to address the challenges posed by this antibiotic-resistant pathogen.

## 1. Introduction

In recent times, the rapid global dissemination of antibiotic-resistant bacteria has become an increasingly pressing issue within the realm of global health [1]. This development has led to a multitude of unfavorable consequences, including a surge in disease occurrence, elevated mortality rates, and a substantial escalation in healthcare expenditures [2]. Among the various microorganisms contributing to this predicament, *Acinetobacter baumannii*, a Gram-negative bacterium, has garnered attention due to its remarkable ability to evolve a diverse range of strategies for resisting antibiotics [3]. Of particular concern is its capability to withstand carbapenems, a class of antibiotics frequently regarded as the last resort in treating infections that are resistant to multiple drugs [4].

The emergence and widespread prevalence of carbapenem-resistant *Acinetobacter baumannii* (CRAB) strains have posed significant challenges in the clinical management of infections. Carbapenems were once highly effective in treating severe infections caused by Gram-negative bacteria. However, the rise of CRAB strains has substantially compromised their efficacy. This has necessitated healthcare providers to turn to alternative antibiotics, often with reduced effectiveness, resulting in prolonged illnesses, increased mortality, and heightened healthcare expenses [5].

To gain a deep and up-to-date comprehension of the complex landscape of scientific research related to the CRAB crisis, bibliometric analysis has emerged as a valuable tool. Bibliometric analysis is a quantitative method that enables the systematic assessment of research outputs, citation patterns, collaborative networks, and the evolutionary path of research articles [6]. In the context of carbapenem-resistant *Acinetobacter baumannii*, the application of bibliometric analysis offers a structured means of extracting crucial insights into the global research scene, pinpointing influential contributors, highlighting significant research milestones, and illuminating emerging research directions [7].

The core objective of this study is to conduct an extensive bibliometric analysis of the international scientific research dedicated to carbapenem-resistant *Acinetobacter baumannii.* By amalgamating data from various sources and employing advanced analytical methods, this analysis aims to unveil intricate patterns of research engagement, collaborative dynamics, publication trends, and thematic focuses within this specialized domain [8]. The ultimate purpose of this study is to provide a comprehensive understanding not only of the current state of knowledge but also of research gaps that require attention. It is expected that the insights derived from this analysis will guide future research priorities and inform strategies to tackle the significant challenges presented by carbapenem-resistant *Acinetobacter baumannii* [9]. Addressing this crisis demands a multifaceted approach encompassing clinical interventions, advancements in research, infection control measures, and public health policies.

## 2. Materials and Methods

### 2.1. Data Collection

The initial phase of our bibliometric analysis involved collecting an extensive dataset of scientific publications concerning carbapenem-resistant *Acinetobacter baumannii* (CRAB); this was performed using the Scopus database on 25 July 2023. We conducted a thorough search across Scopus databases utilizing a combination of relevant keywords such as “carbapenem-resistant”, “*Acinetobacter baumannii*”, “CRAB infections”, and “antibiotic resistance *A. baumannii*” [10]. A further search was limited to the language “English” and the document type “Research Article”, which generated a list of 2415 documents from 1996–2023. The outcomes were extracted and examined for any identical entries utilizing Zotero (version 6.0.23). Following the duplication assessment (which revealed no instances of repetition), a total of 2415 articles were employed for data interpretation, as depicted in Table 1.

### 2.2. Inclusion and Exclusion Criteria

**Figure 1:**
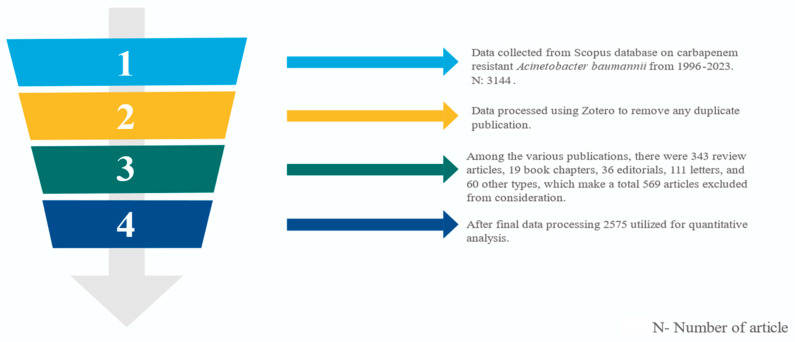
Flowchart representation of articles chosen for quantitative assessment.

We incorporated peer-reviewed journal articles explicitly addressing the topic of carbapenem-resistant *A. baumannii*. To maintain precision, publications concerning different bacterial strains or unrelated subjects were deliberately excluded from the dataset [11]. Further journal articles published in the English language were included in the bibliometric analysis as illustrated in Figure 1.

### 2.3. Data Extraction

For each selected publication we systematically extracted essential details, including title, authorship, publication year, journal source, abstract, keywords, and citation count. This meticulously organized data formed the foundation for the subsequent analytical phases [12].

### 2.4. Bibliometric Analysis

Bibliometric analysis is a method that quantitatively assesses the academic literature to evaluate the influence of research, identify trends, and understand connections. It relies on various indicators such as citation analysis, co-authorship networks, and publication trends. These analytical tools are of great importance to researchers and institutions as they help measure the impact of research findings, pinpoint possibilities for collaboration, and monitor the development of scientific domains. The analysis tools used in this study are VOS viewer [13] and RStudio [14]. VOS viewer version 1.6.19 is used to generate visually compelling representations, including collaboration networks, keyword associations, and publication trends. Further data analysis was conducted using RStudio version 2023.06.1 build 524.

- Publication Trends and Growth: We examined yearly publication trends to discern the evolution of CRAB research over time. Growth rates were calculated and periods of significant expansion were pinpointed [10].
- Authorship and Collaboration: Key contributors and research collectives were identified, with a focus on author collaborations. Collaborative networks were visualized to underscore the relationships between authors and institutions [15].
- Citation Analysis: Citation patterns were meticulously analyzed to unearth highly cited works, influential authors, and seminal contributions that have shaped CRAB discourse [11].
- Keyword Analysis: We conducted an in-depth keyword analysis to spotlight predominant themes and research domains within the CRAB literature. Visualizing co-occurring keywords illuminated thematic connections [16].
- Journal Analysis: The distribution of publications across diverse journals was meticulously scrutinized, bringing to light the prominent publication outlets for CRAB research [17].
- Thematic mapping: Thematic mapping within the realm of bibliometric analysis represents a systematic method for visually comprehending the research terrain of a particular field or subject area. It entails the recognition of fundamental themes, subjects, or research groupings embedded in a corpus of scholarly publications. This systematic approach empowers researchers to delve into and classify the content of these publications, thereby simplifying the process of identifying trends, research trajectories, and the progression of knowledge within the specific domain [18].

## 3. Results and Discussion

Data retrieved from the Scopus database in BibTeX format on 25 July 2023 unveiled a cumulative count of 3144 publications encompassing carbapenem-resistant *Acinetobacter baumannii* spanning the years from 1996 to 2023. Following this retrieval, 343 reviews, 111 letters, 19 book chapters, 36 editorials, and 160 publications categorized as “other” were systematically excluded from our analysis due to their inconsistency with our targeted article classifications, as depicted in Figure 1. Ultimately, 2415 original articles remained eligible for rigorous quantitative analysis. The comprehensive data summary is presented in Table 1 within the RStudio environment. It is worth noting that the computed average annual growth rate of publications in the CRAB research field stands at 21.94%, underscoring the dynamic and progressively expanding nature of this field of study.

### 3.1. Yearly Published Articles in Bibliometric Analysis

**Figure 2:**
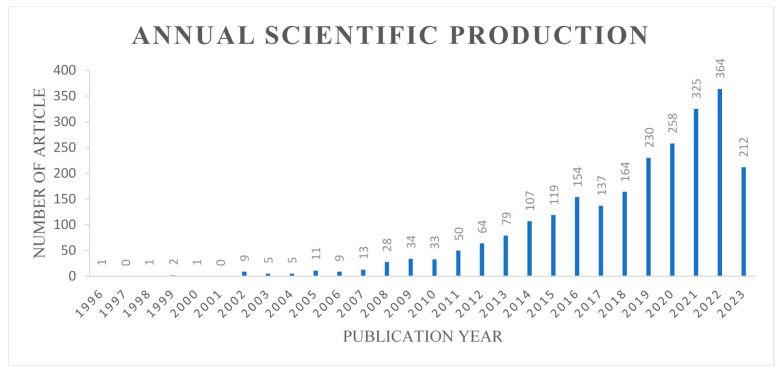
Graph depicting the number of research articles published from 1996 to 2023.

Monitoring the growth of publications in a particular field can serve as a valuable means of comprehending its evolving trends and developments. This analysis not only provides insights into potential research directions but also aids in devising strategies to address emerging challenges [19]. Further investigation and annual assessment of publication numbers can gauge the research trajectory within the expanding domain of traceability in carbapenem-resistant *Acinetobacter baumannii* research (see Figure 2). Our findings revealed that the first article was published in 1996. Subsequently, between 1996 and 2000 only five articles were published, and from 2001 to the end of 2007, a modest 52 articles emerged, averaging fewer than 20 articles annually.

There was a gradual year-on-year increase in the number of publications after 2007. Remarkably, the peak occurred in 2022, when there were 453 articles, closely followed by 2019, 2020, 2021, and 2023, which saw 327, 347, 413, and 268 articles, respectively. Notably, 2023 has already seen the publication of 268 papers. As depicted in the graph, there has been an exponential surge in publications since 2008. Upon closer examination, it becomes increasingly evident that carbapenem-resistant *Acinetobacter baumannii* (CRAB) has emerged as a predominant antibiotic-resistant bacterium of paramount concern that is intimately associated with hospital-acquired infections and therapeutic shortcomings within healthcare facilities [20]. Recent research not only reaffirms the seriousness of this predicament but also illuminates a disquieting development. Specifically, investigations conducted by Maboni and colleagues in 2020 have drawn attention to the existence of *Acinetobacter* species originating in the animal kingdom, characterized by a noteworthy level of resistance to multiple drugs [21]. This discovery not only underscores the disconcerting potential of these pathogens to pose a substantial health hazard but also underscores the likelihood of their proliferation within the human ecosystem. Collectively, these revelations underscore the pressing and intricate nature of the challenges associated with addressing CRAB-related issues, both within the clinical setting and the broader environmental landscape. This unequivocally suggests that the field of carbapenem-resistant *Acinetobacter baumannii* has garnered substantial attention from researchers worldwide, underscoring its emergence as a highly promising research area.

### 3.2. Publication Analysis Based on Geographic Distribution

An extensive review of the publications spanning from 1996 to 2023 was conducted to analyze the worldwide geographic dispersion of research contributions in the field of carbapenem-resistant Acinetobacter baumannii (CRAB). This examination involved categorizing countries based on their publication outputs, offering insight into the degree of their involvement in CRAB research. The leading contributors to this field, based on their publication frequency, were as follows: China (2130), the USA (1308), Italy (628), South Korea (452), and Brazil (381).

**Figure 3:**
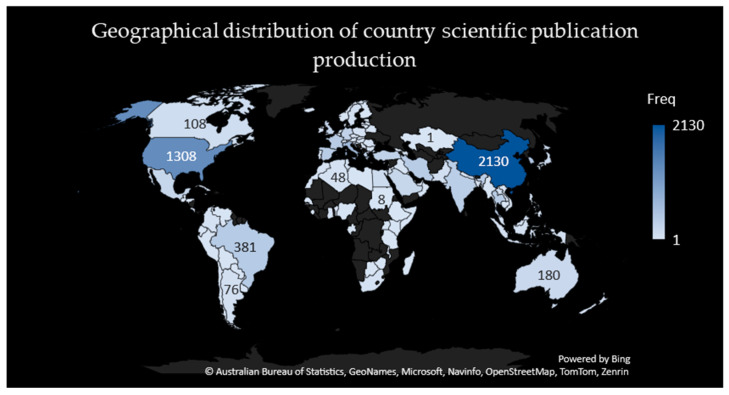
Geographical distribution of the scientific publication production of countries.

For a visual representation of this global distribution, please refer to Figure 3, which showcases a geographical map illustrating the concentration of scientific publications across different countries. This geographic perspective underscores the extensive and diverse participation of nations in CRAB research, highlighting the worldwide significance and collaborative nature of endeavors aimed at addressing this pressing healthcare challenge.

**Figure 4:**
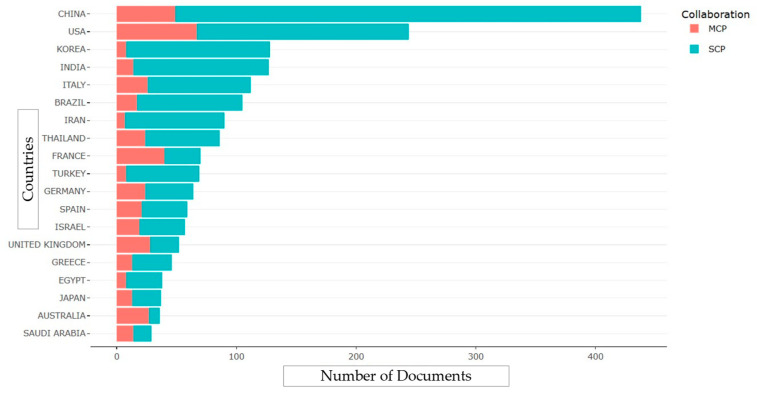
Corresponding author countries based on single-country publications and multiple-country publications.

Furthermore, data analysis of both intra- and inter-country collaborations, focusing on single-country publications (SCPs) and multiple-country publications (MCPs), is shown in Figure 4. The SCP analysis revealed that China has the highest number of SCPs, suggesting that it is primarily engaged in research without extensive international collaboration, despite having the most published articles in the CRAB research field. Conversely, the MCP analysis demonstrated that the USA has a higher number of publications involving multiple countries, indicating a more extensive collaborative network compared with China and other nations.

**Figure 5:**
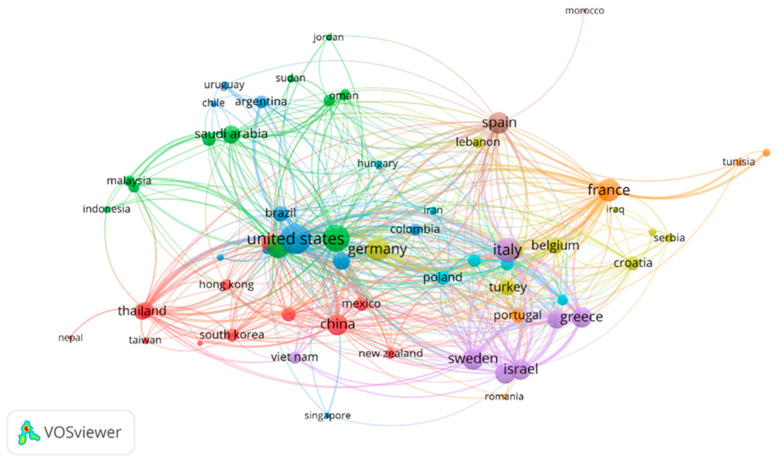
Network representation of collaborative research networks between countries. To be included in this visualization countries were required to have at least five publications in the field under study. The visual presentation employs different colors to signify separate clusters. In this context, a cluster signifies a collection of closely interconnected nodes or entities. Each entity within the network is distinctly assigned to a particular cluster, and the color assigned to it indicates its cluster membership. The lines connecting these entities represent associations or links, with the closeness of two items roughly representing the strength of their connection.

In our pursuit of comprehending collaboration networks, we employed a visualization strategy to assess the interactions between different countries based on total link strength (TLS), as elaborated in Table 2. The total link strength serves as a quantitative measure of the extent to which various entities, including authors or documents, are intricately interconnected within the network. This metric is instrumental in identifying the pivotal and influential constituents within the network, thereby facilitating the evaluation of the robustness of collaborative and intellectual associations. Our analysis, as depicted in Table 2, reveals that China boasts the highest number of articles, totaling 334, with the United States following closely with 311 articles. Nonetheless, it is noteworthy that the total link strength for the United States substantially surpasses that of China, registering at 281 as opposed to China’s 109. This analysis illuminates the paramount role played by the United States in fostering expansive collaborative research efforts within the realm of carbapenem-resistant Acinetobacter baumannii (CRAB) research and highlights the nation’s substantial and impactful engagements with multiple countries in this field. Furthermore, our data show that the USA has the highest number of citations (13,009). To visualize the collaboration networks of countries, we employed VOSviewer, focusing on countries with a minimum of five documents. Out of 136 countries, 63 countries met this threshold and were selected for analysis, as shown in Figure 5.

### 3.3. Bibliometric Analysis of Co-Authorship

Analyzing co-authorship through bibliometric analysis provides valuable insights into the collaborative dynamics within the scientific community. Our data analysis reveals that a total of 6437 authors have contributed papers on carbapenem-resistant *Acinetobacter baumannii* (CRAB). For a more in-depth examination, we employed VOSviewer to scrutinize 112 authors with more than five publications in this field.

Among these prolific authors, Higgins PG, Bonomo RA, Chen Y, LI J, and Wang Y emerged as the top five authors with the highest number of articles in CRAB research (Table 3).

**Figure 6:**
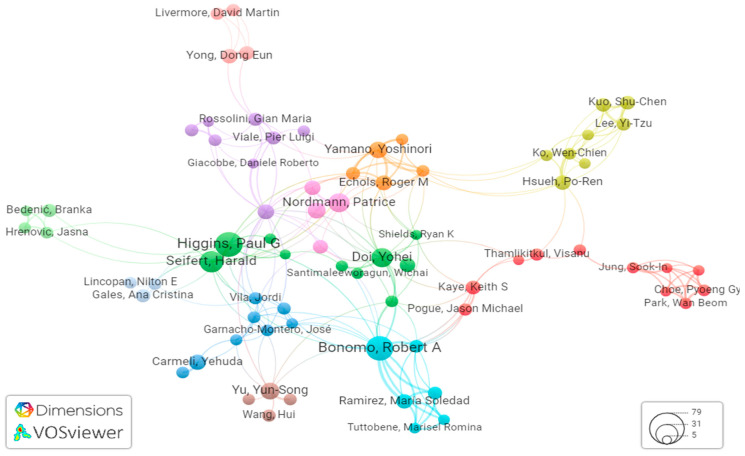
Bibliometric assessment of co-authorship. Colors represent distinct clusters, circle sizes reflect publication counts, and line thickness signifies the intensity of connections.

Notably, Higgins PG from the Institute for Medical Microbiology, Immunology and Hygiene, University of Cologne, Germany stands out with 39 documents and an impressive article fractionalized of 5.76, making him a leading figure in CRAB research. His collaborative network exhibits a robust total link strength of 78 (Figure 6).

**Figure 7:**
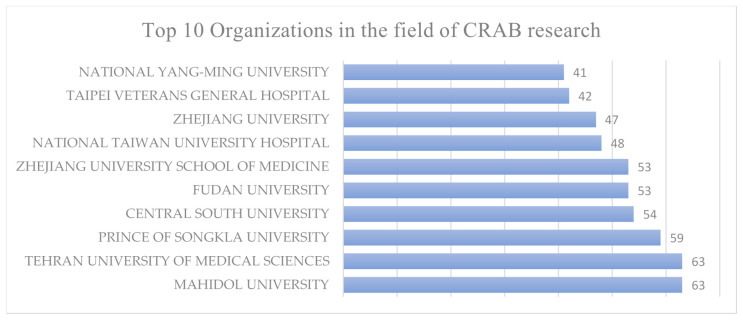
The top 10 institutions and number of articles published by them in the field of CRAB research.

When considering institutions, Mahidol University and Tehran University of Medical Sciences take the lead as the top institutional contributors to CRAB research, with each of them associated with 63 published documents. Further details regarding the top 10 institutional contributors can be found in Figure 7.

**Figure 8:**
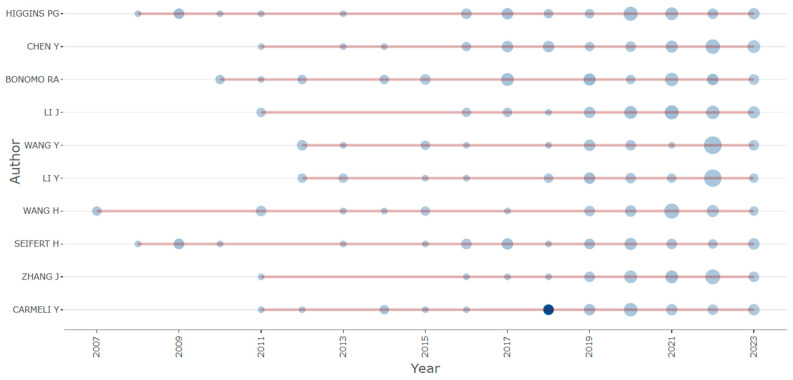
Involvement of the top 10 authors across various years (represented by red lines). The size of the dots corresponds to the number of publications in different years, whereas the color of the dots (ranging from light to dark) signifies the total citations (TC) per year.

In Figure 8, we present the top 20 contributing authors in CRAB research, along with their article publication patterns over the years. This visualization indicates that the publication activity in the CRAB research field commenced in 2011, with most authors beginning their article contributions from that year onwards. It is noteworthy that Higgins, Seifert, and Wang each have approximately five articles published before 2011, indicating their early involvement in this research area.

### 3.4. Bibliometric Analysis of Keyword Co-Occurrence

The analysis of keywords in bibliometric studies serves as a potent tool for revealing and comprehending the fundamental themes, trends, and intellectual framework of a specific research domain. Our dataset, sourced from the Scopus database, underwent an examination of keyword co-occurrence. Out of a total of 3149 identified keywords, those occurring more than five times were categorized as high-frequency keywords and included in our analysis. Consequently, among the 3149 keywords, 264 met this criterion and were incorporated into our analysis.

The most prevalent keyword observed was “*Acinetobacter baumannii*” with 562 occurrences and a substantial total linkage strength of 1320. Following closely was the keyword “carbapenem resistance”, which appeared 196 times and exhibited a total linkage strength of 490 (see Table 4).

**Figure 9:**
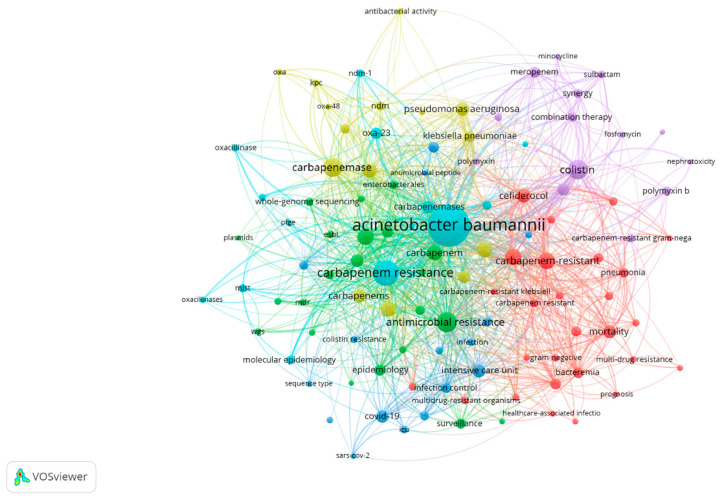
A bibliometric examination of author keywords, emphasizing their co-occurrence. Clusters are denoted by the color, the size of circles signifies the frequency of occurrences, and the thickness of lines represents the strength of connections.

To visually represent the co-occurrence of these keywords, we utilized VOSviewer in Figure 9.

**Figure 10:**
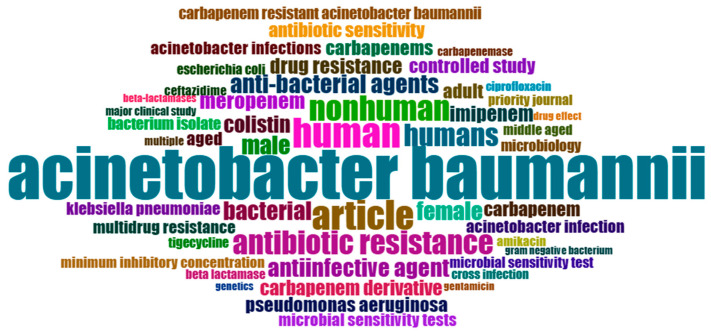
Word cloud generated using RStudio; the size of the word corresponds to its frequency of occurrence.

**Figure 11:**
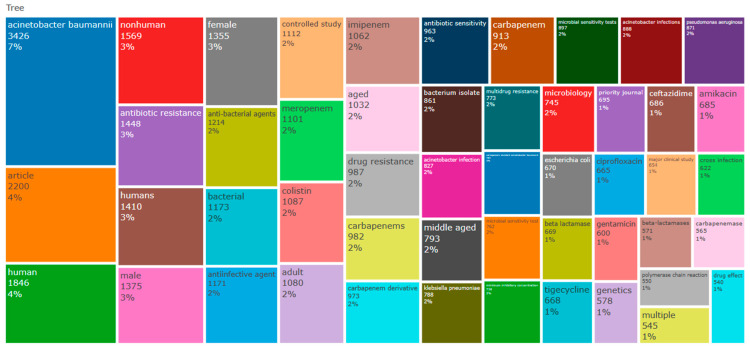
A tree map illustrating the most commonly used words, with percentages indicating the frequency of each keyword’s appearance in the published articles.

Additionally, Figure 10 and Figure 11 present a word cloud and tree map generated using RStudio, highlighting the most frequently occurring keywords.

### 3.5. Bibliometric Analysis of Citations

The utilization of bibliometric analysis for studying citations represents a methodological approach of paramount importance in the realm of academic research and scientific investigation. It functions as a potent instrument for gaining invaluable insights into the sway, significance, and patterns of knowledge dissemination within the scholarly and scientific communities [22].

In our data analysis, we have undertaken the task of ranking the top 10 most-cited documents in the arena of research concerning carbapenem-resistant *Acinetobacter baumannii* (CRAB), as meticulously presented in Table 5. At the zenith of this ranking is the research article authored by Carmeli et al. in 2018 titled “Discovery, Research, and Developments of New Antibiotics: The WHO Priority List of Antibiotics resistant Bacteria and Tuberculosis”, which proudly stands as the most-cited document within the CRAB research domain [23].

Within this landscape of citations, the United States (USA) emerges as the foremost country, boasting a substantial count of 13,983 citations and an impressive average article citation rate of 57.10, as meticulously detailed in Table 6. A close runner-up in this citation race is China, which secures the second-highest citation count of 6027, accompanied by an average article citation rate of 14.00.

In terms of scholarly journals, *The Lancet Infectious Diseases* journal garners notable attention. Despite publishing a mere five articles, it commands the highest number of citations, totaling 3734 citations. On the other hand, the *Antimicrobial Agents and Chemotherapy* journal, with its substantial publication output of 82 articles, secures the second-highest position in terms of citations within the CRAB research sphere, amassing a commendable 2417 citations, as vividly depicted in Table 7.

Additionally, institutional affiliations play a pivotal role in the realm of research citations. The Centre for Anti-Infective Agents in Vienna, Austria, is associated with five articles, yet it garners a commendable 3176 citations. The Institute for Medical Microbiology at the University of Cologne, Germany, emerges as the second-highest affiliated institution within the CRAB research field, boasting an association with 21 articles and garnering a total of 661 citations.

**Figure 12:**
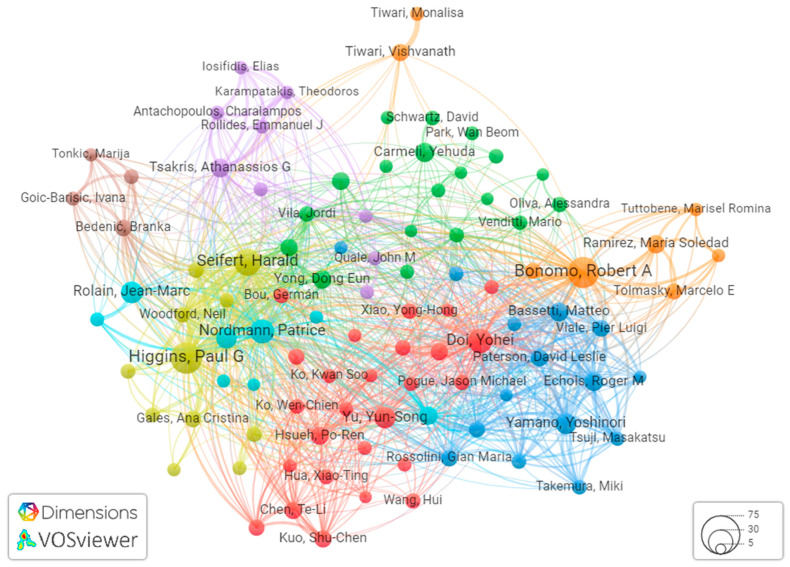
Visualization of the author citation network using VOSviewer. Clusters are identified by distinctive colors, circle sizes correspond to citation counts, and line thickness indicates connection strength.

These findings collectively underscore the profound impact and collaborative synergy within the research community that is dedicated to addressing the multifaceted challenges presented by CRAB. An author network based on citations and visualized using VOSviewer was created. The analysis was limited to a subset of 100 researchers within the network, focusing exclusively on the largest connected set of authors, as depicted in Figure 12. Within the context of this pivotal research domain, distinct documents, nations, academic journals, and institutions have emerged as significant contributors, exerting a lasting influence on the CRAB (carbapenem resistant *Acinetobacter baumannii*) landscape.

### 3.6. Theme Terms and Trend Topic Analysis

In our exploration of thematic mapping, we adopted a graphical approach to illustrate the clusters within the co-occurrence network. This visualization methodology is founded on Callon’s centrality and density ranking system [33]. Within this visual representation, clusters are depicted as bubbles and their dimensions are proportional to the frequency of words contained within them. On the *X*-axis, we portray the centrality of network clusters, denoting the degree of their interactions with other clusters in the graph, thereby signifying the significance of a particular thematic area. On the other hand, the *Y*-axis represents density, which measures a cluster’s internal cohesion and the expansion of its thematic content [34,35,36].

**Figure 13:**
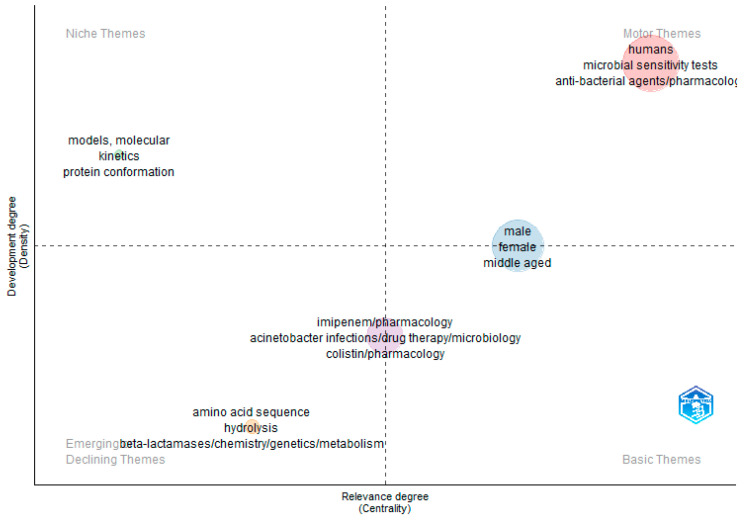
Thematic mapping of theme terms in the field of CRAB research.

This graphical representation yields several noteworthy observations (as mentioned in Figure 13):

(a) Motor Themes (First Quadrant, Top Right): These clusters exhibit high centrality and density, indicating that they are well established and pivotal in shaping the research domain. The cluster highlighted in red within the motor theme comprises terms such as “humans”, “microbial sensitivity”, “antibacterial agents”, and “multi-drug resistance pathogens”. This underscores that the predominant research focus in the CRAB research field revolves around these key concepts.
(b) Niche Themes (Second Quadrant, Top Left): These themes demonstrate high density but low centrality, suggesting that they are of limited relevance and may pertain to specialized or less prominent aspects. The cluster in green within the niche theme encompasses terms such as “molecular kinetics” and “protein conformations”, which have relatively restricted relevance to our research topic.
(c) Emerging or Declining Themes (Third Quadrant, Bottom Left): Clusters located in this quadrant possess low centrality and density, indicating that they are either in their early stages of development or diminishing in importance. The orange cluster within the emerging/declining theme includes terms such as “carbapenem metabolism”, “amino acid sequence”, and “beta-lactamase”. This signifies that certain terms, such as “beta-lactamase”, warrant greater attention, whereas others, such as “amino acid sequences of carbapenemase enzyme”, are in the nascent stages of research within the CRAB research field. Additionally, the second cluster in purple, situated along the central line, exhibits a high degree of relevance. It encompasses terms such as “imipenem”, “meropenem”, “drug resistance”, and “*Acinetobacter* infections”, which are at the core of our research focus within the CRAB research domain.
(d) Basic Themes (Fourth Quadrant, Bottom Right): These themes possess high centrality but low density. They are crucial for addressing transdisciplinary research issues. The blue cluster contains terms such as “intensive care unit”, “antibacterial drug effects”, and “middle-aged” that are primarily centered on research concerning the precautions and measures required in cases of CRAB infection.

Through the utilization of this visual approach, we have effectively delineated the dynamics inherent within thematic clusters, shedding light on their evolutionary trajectories over time. Within this visual representation, the progression of themes over time is revealed by segmenting time into distinct periods. A trajectory moving toward the upper right signifies an ascending trend, indicative of the growing importance and evolution of particular themes. Conversely, a trajectory veering toward the lower left indicates a descending trend, where themes are diminishing in significance. In essence, this analysis underscores that themes related to antibacterial agents and their pharmacology, multi-drug resistance pathogens, and microbial sensitivity are experiencing a pronounced upward trajectory in the CRAB research field.

**Figure 14:**
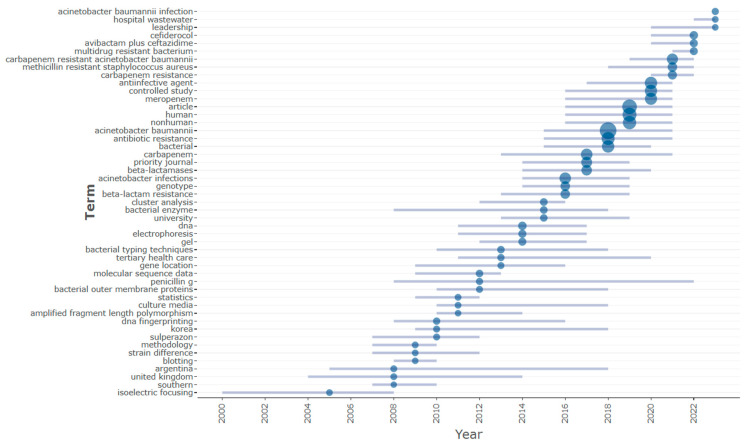
Graphical representation of the most frequently occurring keywords.

Moreover, Figure 14 provides a graphical depiction of the most frequently occurring keywords over the years, forming a trend topic list within the domain of CRAB research. The graph primarily focuses on the last five years, highlighting that “*Acinetobacter baumannii*”, “multi-drug resistance”, “carbapenem resistance”, and “carbapenem-resistant Acinetobacter baumannii” stand out as the most trending topics in CRAB research during this period. These keywords signify the forefront of research endeavors in the CRAB research field, reflecting the current areas of heightened interest and investigation.

## 4. Future Perspective

Prognosis: Comprehensive bibliometric analysis underscores a notable shift in the trajectory of CRAB research, with a growing emphasis on prognostic factors. The alarming mortality rates associated with CRAB infections serve as a poignant reminder of the urgency of deepening our comprehension of prognostic markers [37]. In the years ahead, we anticipate a transformative phase in this domain, characterized by the emergence of personalized prognostic tools. The integration of machine learning algorithms into the analytical framework holds substantial promise. These algorithms are poised to revolutionize clinical practice by enabling the precise prediction of clinical outcomes based on a myriad of patient-specific variables. This transformative approach has the potential to reshape clinical intervention strategies. By identifying key determinants of poor prognosis, such as underlying health conditions and infection sites, clinicians armed with this knowledge can intervene proactively with tailored treatments and intensive care measures. This proactive stance may ultimately yield significant improvements in patient outcomes [38].

Diagnosis: The analysis also highlights a growing preoccupation with advanced diagnostic techniques within the CRAB research sphere. Molecular methodologies, including PCR and next-generation sequencing, have swiftly gained prominence due to their ability to identify resistance mechanisms and pinpoint specific bacterial strains. This trend signifies not only the adoption of cutting-edge technologies but also a broader shift towards precision medicine. We anticipate that future treatment approaches will increasingly hinge on the genetic profiles of CRAB pathogens. Moreover, the emergence of point-of-care diagnostics signals a pivotal step toward decentralized testing, which is particularly vital for resource-constrained settings [39]. Furthermore, in the research landscape there will be a pressing need to streamline and optimize these diagnostic tools further. Such enhancements will be pivotal in ensuring the accurate and timely identification of CRAB infections. This, in turn, will facilitate prompt interventions and serve as a crucial measure to mitigate the risks of disease transmission.

Treatment: The bibliometric analysis indicates a multidimensional approach to CRAB treatment research, reflective of the multifaceted challenges posed by this resilient pathogen [40]. Attention is increasingly gravitating towards combination therapies and the development of innovative drugs designed to counteract resistance mechanisms [41]. Nevertheless, the most noteworthy shift in focus is evident in the exploration of alternative treatment modalities, such as bacteriophage therapy and immune-modulating agents. These initiatives underscore the urgency of finding solutions that extend beyond conventional antibiotic paradigms. This hints at a potential paradigm shift, where the spotlight may transition from solely targeting the pathogen to bolstering the host’s immune response [42]. Furthermore, the integration of nanotechnology-based drug delivery systems underscores the evolving precision medicine approach [43,44]. This innovative avenue seeks to enhance treatment efficacy while simultaneously minimizing the undesirable side effects associated with broad-spectrum antibiotics.

The collaborative nature of ongoing research efforts stands as a testament to the collective determination within the scientific community. This spirit of collaboration signals a concerted effort to devise innovative strategies through interdisciplinary cooperation. This collaborative ethos promises an exciting and dynamic future in the relentless pursuit of effective CRAB infection treatments. It offers hope for improved patient outcomes and a more secure healthcare landscape.

## 5. Limitations

Database Selection: This study exclusively relied on the Scopus database, mainly due to its extensive coverage and the inclusion of cited references, which are essential for conducting thorough bibliometric analysis. However, depending solely on a single database introduces the possibility of biases related to coverage and indexing practices, as alternative databases may produce slightly different outcomes [45,46].

Coverage Bias: Despite Scopus’s comprehensive nature, there is the potential for some publications to be overlooked. Older publications, non-English articles, or research in specialized fields that are not well-represented in Scopus might not be fully encompassed, potentially impacting the comprehensiveness of the study [47].

Data Accuracy: Ensuring the precision of bibliometric data holds the utmost importance. Errors or inconsistencies in author names, affiliations, publication titles, or citation counts within the database have the potential to influence the credibility of the analysis. Although diligent efforts were made to minimize inaccuracies, it is worth acknowledging that some may still exist within the Scopus dataset.

Publication Lag: Delays in the incorporation of research into databases such as Scopus can affect the timeliness of the analysis. Recent developments or newly published works might not be entirely integrated, potentially influencing the study’s currentness.

Language Bias: The decision to restrict the analysis to research articles published in English introduces the possibility of language bias. This limitation could result in the exclusion of valuable contributions published in languages other than English, potentially impacting the study’s comprehensiveness [47].

Search String Limitations: Formulating robust search queries for bibliometric analysis presents inherent challenges. Despite meticulous efforts to create comprehensive search strings, it is important to acknowledge that no search string is flawless. Some relevant publications may have been inadvertently omitted, whereas irrelevant ones may have been included in the analysis.

It is imperative to bear these limitations in mind when interpreting the findings of this bibliometric study. It is important to recognize that Scopus served as the primary data source and that measures were implemented to enhance data accuracy. Nonetheless, these constraints have the potential to influence the study’s scope and results.

## 6. Conclusions

The escalating challenge posed by carbapenem-resistant *Acinetobacter baumannii* (CRAB) amplifies the worldwide need for a deeper and thorough scientific exploration, especially considering the pathogen’s increasing resilience and associated health threats. Spanning from 1996 to 2023, our analysis delved into 2415 primary articles from an aggregate of 3144 pieces sourced from the Scopus database. In recent years, keywords such as “*Acinetobacter baumannii*”, “multi-drug resistance”, “carbapenem resistance” and “carbapenem-resistant *Acinetobacter baumannii*” have prominently figured in CRAB studies, signaling evolving interests and mounting apprehensions in the field. China’s dominance in publishing and the USA’s influential position, as gauged by citations, highlight the global resonance of this matter. The noteworthy contributions of Higgins PG, evidenced by his 39 key papers, exemplify the vital impact individual scholars have in directing this research area. Employing a bibliometric approach not only sheds light on historical research activities but also aids in identifying areas of concern, discerning novel trends, and guiding subsequent investigative paths. In the face of the deepening CRAB predicament, a comprehensive assessment such as this accentuates the continuous and collective global push required to address the issues brought forth by this resistant strain.

## Figures and Tables

**Table 1:** Overview of gathered data.

Data Aspects	Statistical Finding
Data Overview	
Time Range	1996 to 2023
Sources (Journals, Books, etc.)	573
Total Documents	2415
Average Annual Publication Growth Rate %	21.94
Average Age of Documents	4.9
Average Citations per Document	25.38
Document Contents	
Keywords Plus (ID)	9324
Author’s Provided Keywords (DE)	3388
Authorship	
Total Authors	
Authors of Sole-Authored Documents	24
Authors Collabortion	
Sole-Authored Documents	24
Co-Authors per Document	8.19
International Co-Authorships %	24.1
Document Types	
Article	2415

**Table 2:** Top countries publishing the most articles in the field of CRAB based on total link strength.

Country	Research Articles		Citations		Total Link Strength (TLS)	
	Number of Research Articles	Rank	Number of Citations	Rank	TLS	Rank
Australia	56	12	4555	6	136	5
Austria	20	19	3496	12	86	13
China	334	1	3996	9	109	9
France	81	8	4729	5	135	6
Germany	101	5	5238	3	144	4
Greece	53	15	1722	15	104	11
India	126	3	1786	14	65	16
Israel	58	11	4479	7	102	12
Italy	124	4	6079	2	168	3
Japan	54	14	2511	12	58	18
Netherlands	31	18	3761	11	106	10
Saudi Arabia	55	13	840	18	71	15
Spain	75	9	1901	13	119	7
Sweden	34	16	4209	8	114	8
Switzerland	34	17	3794	10	79	14
Thailand	88	6	986	17	64	17
Turkey	64	10	1295	16	58	19
United Kingdom	85	7	4852	4	196	2
United States	311	2	13,009	1	281	1

**Table 3:** Most productive authors in the field of CRAB research.

Authors	Articles	Articles Fractionalized
Higgins PG	39	5.76
Bonomo RA	37	4.79
Chen Y	37	3.99
Li J	36	3.56
Wang Y	35	3.63
Li Y	34	3.93
Wang H	34	4.89
Seifert H	32	4.83
Zhang J	32	3.25
Carmeli Y	31	4.04

**Table 4:** Top 10 most frequently occurring keywords.

S No.	Keyword	Occurrence	Total Link Strength
1	*Acinetobacter baumannii*	532	1320
2	Carbapenem resistance	196	490
3	Antimicrobial resistance	121	277
4	Colistin	115	332
5	Carbapenemase	107	284
6	Carbapenem-resistant	86	233
7	Antibiotic resistance	80	187
8	Carbapenem-resistant *Acinetobacter baumannii*	78	155
9	Gram-negative bacteria	72	193
10	Multidrug resistance	71	198

**Table 5:** The ranking of the ten most-cited documents in the CRAB research field.

Title	Journals	Total Citations	Reference
“Discovery, research, and development of new antibiotics: the WHO priority list of antibiotic-resistant bacteria and tuberculosis”	*The Lancet Infectious Diseases*	4000	[23]
“Global spread of carbapenem-resistant *Acinetobacter baumannii*”	*Journal of Antimicrobial Chemotherapy*	822	[24]
“Colistin alone versus colistin plus meropenem for treatment of severe infections caused by carbapenem-resistant Gram-negative bacteria: an open-label, randomized controlled trial”	*The Lancet Infectious Diseases*	461	[25]
“OXA-143, a Novel Carbapenem-Hydrolyzing Class D β-Lactamase in *Acinetobacter baumannii”*	*Antimicrobial Agents and Chemotherapy*	298	[26]
“Molecular Epidemiology of Clinical Isolates of Carbapenem-Resistant *Acinetobacter* spp. from Chinese Hospitals”	*Antimicrobial Agents and Chemotherapy*	279	[27]
“New Treatment Options against Carbapenem-Resistant *Acinetobacter baumannii* Infections”	*Antimicrobial Agents and Chemotherapy*	266	[28]
“Carbapenem-resistant *Acinetobacter baumannii* and Klebsiella pneumoniae across a hospital system: impact of post-acute care facilities on dissemination”	*Journal of Antimicrobial Chemotherapy*	245	[29]
“Combination therapy for carbapenem-resistant Gram-negative bacteria”	*Journal of Antimicrobial Chemotherapy*	247	[30]
“Active and Passive Immunization Protects against Lethal, Extreme Drug Resistant-*Acinetobacter baumannii* Infection”	*PLoS ONE*	195	[31]
“The antimicrobial peptide ZY4 combats multidrug-resistant *Pseudomonas aeruginosa* and *Acinetobacter baumannii* infection”	*Proceedings of the National Academy of Sciences*	189	[32]

**Table 6:** Leading nations by citations and their average citations per article.

Country	TC	Average Article Citations
USA	13,009	57.10
CHINA	6027	14.00
GERMANY	4787	74.80
UNITED KINGDOM	3463	67.90
SPAIN	2760	47.60
FRANCE	2568	36.70
KOREA	2419	19.00
ITALY	2259	20.40
INDIA	2063	16.40
ISRAEL	1778	31.20

**Table 7:** Highly referenced journals in the CRAB research domain.

S No.	Sources	Documents	Impact Factor	Citations
1	*The Lancet Infectious Diseases*	5	56.3	3734
2	*Antimicrobial Agents and Chemotherapy*	82	4.9	2417
3	*Clinical Infectious Diseases*	34	20.9	2293
4	*Frontiers in Microbiology*	61	6.06	1340
5	*Journal of Antimicrobial Chemotherapy*	50	5.75	1269
6	*Microbial Drug Resistance*	70	2.6	1138
7	*International Journal of Antimicrobial Agents*	52	15.44	964
8	*Journal of Infectious Diseases*	5	7.75	938
9	*Antimicrobial Resistance and Infection Control*	40	5.29	835
10	*PLoS ONE*	37	3.7	818

## Data Availability

Raw data is available from the corresponding author on request.

## References

[1] Aslam B., Wang W., Arshad M.I., Khurshid M., Muzammil S., Rasool M.H., Nisar M.A., Alvi R.F., Aslam M.A., Qamar M.U. (2018). Antibiotic resistance: A rundown of a global crisis. Infect. Drug Resist..

[2] Fazal F., Saleem T., Rehman M.E.U., Haider T., Khalid A.R., Tanveer U., Mustafa H., Tanveer J., Noor A. (2022). The rising cost of healthcare and its contribution to the worsening disease burden in developing countries. Ann. Med. Surg..

[3] Howard A., O’donoghue M., Feeney A., Sleator R.D. (2012). *Acinetobacter* *baumannii*. Virulence.

[4] Almaghrabi M.K., Joseph M.R.P., Assiry M.M., Hamid M.E. (2018). Multidrug-Resistant *Acinetobacter baumannii*: An Emerging Health Threat in Aseer Region, Kingdom of Saudi Arabia. Can. J. Infect. Dis. Med. Microbiol. = J. Can. Des. Mal. Infect. Microbiol. Med..

[5] Wu H.-J., Xiao Z.-G., Lv X.-J., Huang H.-T., Liao C., Hui C.-Y., Xu Y., Li H.-F. (2023). Drug-resistant *Acinetobacter baumannii*: From molecular mechanisms to potential therapeutics (Review). Exp. Ther. Med..

[6] Wang R., Zhu Y., Qin L.-F., Xu Z.-G., Gao X.-R., Liu C.-B., Xu G.-T., Chen Y.-Z. (2023). Comprehensive Bibliometric Analysis of Stem Cell Research in Alzheimer’s Disease from 2004 to 2022. Dement. Geriatr. Cogn. Disord..

[7] Zhu Y., Zhang X., Wang Y., Tao Y., Shao X., Li Y., Li W. (2022). Insight into carbapenem resistance and virulence of *Acinetobacter baumannii* from a children’s medical centre in eastern China. Ann. Clin. Microbiol. Antimicrob..

[8] Huang H.-L., Li Y.-Y., Guo H.-B. (2023). Detection and homology analysis of carbapenem resistant *Acinetobacter baumannii* resistance gene. Front. Cell. Infect. Microbiol..

[9] Jiang Y., Ding Y., Wei Y., Jian C., Liu J., Zeng Z. (2022). Carbapenem-resistant *Acinetobacter baumannii*: A challenge in the intensive care unit. Front. Microbiol..

[10] Chen C. (2017). Science Mapping: A Systematic Review of the Literature. J. Data Inf. Sci..

[11] Waltman L., van Eck N.J. (2012). A new methodology for constructing a publication-level classification system of science: A New Methodology for Constructing a Publication-Level Classification System of Science. J. Am. Soc. Inf. Sci. Technol..

[12] Bornmann L., Haunschild R. (2017). Does evaluative scientometrics lose its main focus on scientific quality by the new orientation towards societal impact?. Scientometrics.

[13] Williams B. (2020). Dimensions & VOSViewer Bibliometrics in the Reference Interview. Code4Lib J..

[14] Aria M., Cuccurullo C. (2017). bibliometrix: An R-tool for comprehensive science mapping analysis. J. Informetr..

[15] Rafols I., Meyer M. (2010). Diversity and network coherence as indicators of interdisciplinarity: Case studies in bionanoscience. Scientometrics.

[16] Leydesdorff L., Rafols I. (2012). Interactive overlays: A new method for generating global journal maps from Web-of-Science data. J. Inf..

[17] Milojević S. (2010). Power law distributions in information science: Making the case for logarithmic binning. J. Am. Soc. Inf. Sci. Technol..

[18] Parlina A., Ramli K., Murfi H. (2020). Theme Mapping and Bibliometrics Analysis of One Decade of Big Data Research in the Scopus Database. Information.

[19] Magadán-Díaz M., Rivas-García J.I. (2022). Publishing Industry: A Bibliometric Analysis of the Scientific Production Indexed in Scopus. Publ. Res. Q..

[20] Douraghi M., Kenyon J.J., Aris P., Asadian M., Ghourchian S., Hamidian M. (2020). Accumulation of Antibiotic Resistance Genes in Carbapenem-Resistant *Acinetobacter baumannii* Isolates Belonging to Lineage 2, Global Clone 1, from Outbreaks in 2012–2013 at a Tehran Burns Hospital. mSphere.

[21] Maboni G., Seguel M., Lorton A., Sanchez S. (2020). Antimicrobial resistance patterns of Acinetobacter spp. of animal origin reveal high rate of multidrug resistance. Vet. Microbiol..

[22] Elango B., Ho Y.-S. (2017). A Bibliometric Analysis of Highly Cited Papers from India in Science Citation Index Expanded. Curr. Sci..

[23] Tacconelli E., Carrara E., Savoldi A., Harbarth S., Mendelson M., Monnet D.L., Pulcini C., Kahlmeter G., Kluytmans J., Carmeli Y. (2018). Discovery, research, and development of new antibiotics: The WHO priority list of antibiotic-resistant bacteria and tuberculosis. Lancet Infect. Dis..

[24] Higgins P.G., Dammhayn C., Hackel M., Seifert H. (2010). Global spread of carbapenem-resistant *Acinetobacter baumannii*. J. Antimicrob. Chemother..

[25] Paul M., Daikos G.L., Durante-Mangoni E., Yahav D., Carmeli Y., Benattar Y.D., Skiada A., Andini R., Eliakim-Raz N., Nutman A. (2018). Colistin alone versus colistin plus meropenem for treatment of severe infections caused by carbapenem-resistant Gram-negative bacteria: An open-label, randomised controlled trial. Lancet Infect. Dis..

[26] Higgins P.G., Poirel L., Lehmann M., Nordmann P., Seifert H. (2009). OXA-143, a Novel Carbapenem-Hydrolyzing Class D β-Lactamase in *Acinetobacter baumannii*. Antimicrob. Agents Chemother..

[27] Wang H., Guo P., Sun H., Wang H., Yang Q., Chen M., Xu Y., Zhu Y. (2007). Molecular Epidemiology of Clinical Isolates of Carbapenem-Resistant *Acinetobacter* spp. from Chinese Hospitals. Antimicrob. Agents Chemother..

[28] Isler B., Doi Y., Bonomo R.A., Paterson D.L. (2019). New Treatment Options against Carbapenem-Resistant *Acinetobacter baumannii* Infections. Antimicrob. Agents Chemother..

[29] Perez F., Endimiani A., Ray A.J., Decker B.K., Wallace C.J., Hujer K.M., Ecker D.J., Adams M.D., Toltzis P., Dul M.J. (2010). Carbapenem-resistant *Acinetobacter baumannii* and Klebsiella pneumoniae across a hospital system: Impact of post-acute care facilities on dissemination. J. Antimicrob. Chemother..

[30] Paul M., Carmeli Y., Durante-Mangoni E., Mouton J.W., Tacconelli E., Theuretzbacher U., Mussini C., Leibovici L. (2014). Combination therapy for carbapenem-resistant Gram-negative bacteria. J. Antimicrob. Chemother..

[31] Luo G., Lin L., Ibrahim A.S., Baquir B., Pantapalangkoor P., Bonomo R.A., Doi Y., Adams M.D., Russo T.A., Spellberg B. (2012). Active and Passive Immunization Protects against Lethal, Extreme Drug Resistant-*Acinetobacter baumannii* Infection. PLoS ONE.

[32] Mwangi J., Yin Y., Wang G., Yang M., Li Y., Zhang Z., Lai R. (2019). The antimicrobial peptide zy4 combats multidrug-resistant pseudomonas aeruginosa and acinetobacter baumannii infection. Proc. Natl. Acad. Sci. USA.

[33] Callon M., Courtial J.P., Laville F. (1991). Co-word analysis as a tool for describing the network of interactions between basic and technological research: The case of polymer chemsitry. Scientometrics.

[34] Cahlik T. (2000). Comparison of the Maps of Science. Scientometrics.

[35] Cobo M.J., López-Herrera A.G., Herrera-Viedma E., Herrera F. (2011). An approach for detecting, quantifying, and visualizing the evolution of a research field: A practical application to the Fuzzy Sets Theory field. J. Informetr..

[36] Alkhammash R. (2023). Bibliometric, network, and thematic mapping analyses of metaphor and discourse in COVID-19 publications from 2020 to 2022. Front. Psychol..

[37] Hamidian M., Nigro S.J. (2019). Emergence, molecular mechanisms and global spread of carbapenem-resistant *Acinetobacter baumannii*. Microb. Genom..

[38] Lee Y.-L., Ko W.-C., Hsueh P.-R. (2023). Geographic patterns of *Acinetobacter baumannii* and carbapenem resistance in the Asia-Pacific Region: Results from the Antimicrobial Testing Leadership and Surveillance (ATLAS) program, 2012–2019. Int. J. Infect. Dis..

[39] Zeng M., Xia J., Zong Z., Shi Y., Ni Y., Hu F., Chen Y., Zhuo C., Hu B., Lv X. (2023). Guidelines for the diagnosis, treatment, prevention and control of infections caused by carbapenem-resistant gram-negative bacilli. J. Microbiol. Immunol. Infect..

[40] Piperaki E.-T., Tzouvelekis L.S., Miriagou V., Daikos G.L. (2019). Carbapenem-resistant *Acinetobacter baumannii*: In pursuit of an effective treatment. Clin. Microbiol. Infect..

[41] Ranjbar R., Zayeri S., Mirzaie A. (2020). Development of multiplex PCR for rapid detection of metallo-β-lactamase genes in clinical isolates of *Acinetobacter baumannii*. Iran. J. Microbiol..

[42] Shields R.K., Paterson D.L., Tamma P.D. (2023). Navigating Available Treatment Options for Carbapenem-Resistant *Acinetobacter baumannii-calcoaceticus* Complex Infections. Clin. Infect. Dis..

[43] Kumar R., Islam T. (2022). Nurunnabi Mucoadhesive carriers for oral drug delivery. J. Control. Release.

[44] Kumar R., Mahapatra S.S., Ranjan S., Dasgupta S., Mishra R.K., Thomas S. (2019). Lipid-based nanoparticles for drug-delivery systems. Nanocarriers for Drug Delivery.

[45] Stasi A., Mir T.U.G., Pellegrino A., Wani A.K., Shukla S. (2023). Forty years of research and development on forensic genetics: A bibliometric analysis. Forensic Sci. Int. Genet..

[46] Sweileh W.M., Al-Jabi S.W., Zyoud S.H., Sawalha A.F., Abu-Taha A.S. (2018). Global research output in antimicrobial resistance among uropathogens: A bibliometric analysis (2002–2016). J. Glob. Antimicrob. Resist..

[47] Zhong H., Chen F., Li Y.-J., Zhao X.-Y., Zhang Z.-L., Gu Z.-C., Yu Y.-T. (2021). Global trends and hotspots in research of carbapenem-resistant Enterobacteriaceae (CRE): A bibliometric analysis from 2010 to 2020. Ann. Palliat. Med..

